# Platinum-Catalyzed Allylation of 2,3-Disubstituted Indoles with Allylic Acetates

**DOI:** 10.3390/molecules22122097

**Published:** 2017-11-29

**Authors:** Bai-Jing Peng, Wen-Ting Wu, Shyh-Chyun Yang

**Affiliations:** 1 School of Pharmacy, College of Pharmacy, Kaohsiung Medical University, Kaohsiung 807, Taiwan; nick4780664@gmail.com (B.-J.P.); jillj6jp6wn6@gmail.com (W.-T.W.); 2 Department of Fragrance and Cosmetic Science, College of Pharmacy, Kaohsiung Medical University, Kaohsiung 807, Taiwan; 3 Department of Medical Research, Kaohsiung Medical University Hospital, Kaohsiung 807, Taiwan

**Keywords:** platinum-catalyzed, indole derivatives, allylic acetates, allylation

## Abstract

Given the importance of heterocycle indole derivatives, much effort has been directed toward the development of methods for functionalization of the indole nucleus at N1 and C3 sites. Moreover, the platinum-catalyzed allyation of nucleophiles was an established and efficient way, which has been applied to medicinal and organic chemistry. In our research, the platinum-catalyzed 2,3-disubstitued indoles with allylic acetates was investigated under different conditions. Herein, we established a simple, convenient, and efficient method, which afforded high yield of allylated indoles.

## 1. Introduction

The asymmetric synthesis of indoles is of great interest because of their prevalent structure motifs in natural products, organic materials, and medicinal compounds [1,2,3]. Especially with indoles and indole-derived heterocycles bearing a 2,3-disubstituted structure in pharmaceutical drugs [4,5,6,7,8]. The 2,3-disubstituted indoles serve as an ambient nucleophile, and some sophisticated conditions are required to achieve selective alkylation either at the N1 or C3 position [9,10,11]. Therefore, given the prevalence of the indole nucleus in biologically active compounds, the direct N1 or C3 functionalization of 2,3-disubstituted indoles represents an important issue [12,13]. Moreover, a particularly difficult transformation is the electrophilic attack at N1 or C3 on 2,3-disubstituted indoles to produce indolenines containing a new C–C or C–N bond [14,15,16]. For these reasons, extensive efforts have been undertaken to explore the catalytic allylation of indoles at N1 or C3 sites [17,18,19,20,21,22,23,24,25].

The allylation reaction of transition metal-catalyzed continue to enjoy increasing popularity as these can be based on a variety of metals and demonstrate regioselectivity to branched isomers [26,27,28,29]. Transition metal η^3^-allyl complexes, as well as transition metal σ-alkyl complexes, play important roles as active species and key intermediates in many reactions with metal-catalyzed system [30]. The palladium-catalyzed allylation is a powerful tool for C–C, C–N, and C–O bond formations, which have been widely applied to organic chemistry [31,32,33,34,35,36]. The processes have been shown to proceed by attack of nucleophiles on intermediate η^3^-allylpalladium (II) complexes generated by oxidative addition of allylic compounds including halides, esters, carbonates, carbamates, phosphates, and related derivatives to a Pd(0) complex [37,38,39,40,41,42,43,44,45,46,47,48,49,50,51,52,53,54]. The palladium and ruthenium had been used in the allylation reaction of 2,3-disubstituted indoles, but according to our knowledge, platinum would not be reported [55,56,57]. The platinum is also a tool for transition metal, which is not often discussed in the reaction of allylation [58,59]. In the past course of our studies by using platinum allylation, we established the application of a processed platinum catalysis with satisfied data [60,61]. Herein, we report a novel catalysis of a platinum complex, which mediates N1-allylation or C3-allylation of 2,3-disubstituted indoles with allylic acetates.

## 2. Results and Discussion

**Scheme 1:**
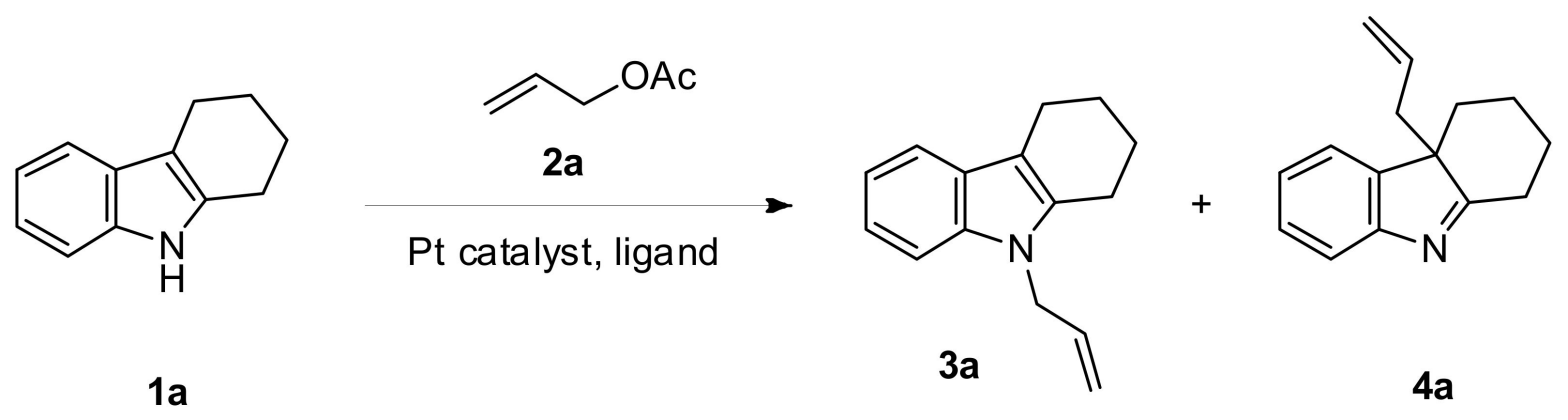
Allylation of 1,2,3,4-tetrahydrocarbazole (**1a**) with allyl acetate (**2a**).

The platinum-catalyzed allylation of 2,3-disubstituted indoles such as 1,2,3,4-tetrahydrocarbazole with allyl acetate was investigated under various conditions (Scheme 1). When a mixture of 1,2,3,4-tetrahydrocarbazole (**1a**, 1 mmol) and allyl acetate (**2a**, 2 mmol) was refluxed in the presence of catalytic amounts of Pt(acac)_2_ (2.5 mmol%) and PPh_3_ (10 mmol%) in benzene for 24 h, *N*-allyl-1,2,3,4-tetrahydrocarbazole (**3a**) was formed in only 7% yield (entry 1 in Table 1). Among the monodentate ligands including PPh_3_ (entry 1), (2-CH_3_C_6_H_4_)_3_P (entry 2), (3-CH_3_C_6_H_4_)_3_P (entry 3), (4-CH_3_C_6_H_4_)_3_P (entry 4), (4-FC_6_H_4_)_3_P (entry 5), (4-ClC_6_H_4_)_3_P (entry 6), (*n*-butyl)_3_P (entry 7), (3-CH_3_OC_6_H_4_)_3_P (entry 8), (4-CH_3_OC_6_H_4_)_3_P (entry 9), (2-furyl)_3_P (entry 10), (2,6-diCH_3_OC_6_H_3_)_3_P (entry 11), and (2,4,6-triCH_3_OC_6_H_2_)_3_P (entry 12) were used. Furthermore, the bidentate ligands dppm (entry 13), dppf (entry 14), dppe (entry 15), and dppb (entry 16) were evaluated in the reaction. The catalytic reactivity of the ligand (4-ClC_6_H_4_)_3_P was likely due to improved catalyst stability and got N-allylation product **3a** and C3-allylation product 4*a*-allyl-2,3,4,4*a*-tetrahydro-1*H*-carbazole (**4a**) in 69 and 30% yields, respectively (entry 6). The regisoselectivity N-allylation and C-allylation of 1,2,3,4-tetrahydrocarbazole was about 2:1 ratio. In our condition, N-allylation of 1,2,3,4-tetrahydrocarbazole was the major compound. The predominant N-allylation derivative might be the result of different elements which seemed to control the reaction; however, no decisive conclusion seemed to have been reached. The reaction did not occur in the absence of the phosphine ligand (entry 17) or platinum species (entry 18). The environmental condition was also investigated. At 50 °C, in the presence of Pt(acac)_2_ and (4-ClC_6_H_4_)_3_P afforded the yields only 11% (entry 19). The reaction gave 45% yields under reflux for 12 h (entry 20). In the presence of various platinum catalysts, including Pt(acac)_2_ (entry 6), *cis*-PtCl_2_(PhCN)_2_ (entry 21), *cis*-PtCl_2_(PPh_3_)_2_ (entry 22), di(1,5-cyclooctadiene)Pt (entry 23), O[Si(CH_3_)_2_C=CH_2_]_2_Pt (entry 24), PtCl_2_ (entry 25), PtI_2_ (entry 26), Pt(CN)_2_ (entry 27), Pt(CH_2_=CH_2_)(PPh_3_)_2_ (entries 28 and 29), and Pt(PPh_3_)_4_ (entries 30 and 31) showed that the most effective platinum catalyst is the Pt(acac)_2_ (entry 6). However, using Pt(CH_2_=CH_2_)(PPh_3_)_2_ or Pt(PPh_3_)_4_ with extra (4-ClC_6_H_4_)_3_P as catalyst increased the yields of products (entries 29 and 31). During the reaction, adding the phosphine ligands could increase the activity of the platinum catalyst. Reduction in the ratio of Pt(acac)_2_ to (4-ClC_6_H_4_)_3_P as 1:1 (entry 32), 1:2 (entry 33), and 1:3 (entry 34) ratios decreased the yield in the reaction. It was known that several factors, such as the solvent and nature of the nucleophile, could alter the product pattern in the metal-catalyzed allylation. The six solvents were investigated (entries 6 and 35–39). Although the toxicity of benzene is known, it was the best solvent in this reaction. This survey defined simple and convenient catalyst way for N-allylation and C-allylation of hindered indoles in high yields (entry 6).

The high efficiency of the allylation reactions described above encouraged us to extend the reaction to allylic compounds. The results for allylation of a number of allylic compounds **2b**–**f** with 1,2,3,4-tetrahydrocarbazole (**1a**) using Pt(acac)_2_ and (4-ClC_6_H_4_)_3_P were summarized in Table 2. 3-Buten-2-yl acetate (**2b**) which reacted with **1a** gave **3b** and **4b** in 54 and 23% yields, respectively (entry 1). The *E*/*Z* ratio of **3b** and **4b** was determined by GC. The product *E* alkene generated from the more thermodynamically stable *syn* complex. The corresponding reaction with crotyl acetate (**2c**) afforded N-allylated and C-allylated tetrahydrocarbazole in overall 87% yields (entry 2). These products might all be derived from the same π-allylic intermediate which could be attacked at the C-1. The reaction was considered to proceed via π-allylplatinum intermediates. The loss of stereochemistry of starting acetate **2b** was due to a rapid σ↔η^3^↔σ interconversion of the intermediates compared to the rate of allylation. Allylation of *trans*-2-hexen-1-yl acetate (**2d**) gave mixtures of N-allylated and C-allylated tetrahydrocarbazole **3c** and **4c** in yields of 45 and 27%, respectively (entry 3). Reaction of allyl chloride (**2e**) produced **3a** and **4a** in yields of 34 and 12%, respectively (entry 4). The allyl chloride is not a good reagent for allylation, but with Pt(acac)_2_ (0.05 mmol) and (4-ClC_6_H_4_)_3_P (0.2 mmol), the totally yields increased to 75% (entry 5). Lastly, reaction of **1a** with allyl carbonate (**2f**) afforded **3a** and **4a** in overall 92% yields (entry 6).

The reaction conditions developed above were found to be useful and efficient to corresponding indole derivatives (Table 3). The results collected in Table 3 shown that allylation of allyl acetate (**2a**) with indoles using Pt(acac)_2_ and (4-ClC_6_H_4_)_3_P, giving general good yields of the corresponding allylic indoles (entries 1–3). Cycloheptane-fused indole (**1b**) was used in the reaction and gave 97% yields of the corresponding N-allylated and C-allylated products (entry 1). 2,3-Dimethyl indole (**1c**) was under investigation. The overall yield was 98% (entry 2). Finally, allylation of the simpler 3-methylindole (**1d**) was tested in the reaction. 3-Methylindole (**1d**) generated the allylation products **3f** in a 47% yield and **4f** in a 24% yield after 24 h (entry 3).

## 3. Experimental Section

### 3.1. General Considerations

Reagents were obtained from Acros Organics (Geel, Belgium), Tokyo Chemical Industry (Tokyo, Japan), Sigma-Aldrich (St. Louis, MO, USA), and Alfa-Aesar (Ward Hill, MA, USA), and used without further purification. All reactions were carried out under a nitrogen atmosphere. Solvents were dried and distilled by known methods. Cloumn chromatography was performed on silica gel. IR absorption spectra were recorded on Shimadzu IR-27G and Perkin-Elmer System 2000FT-IR spectrophotometers. Proton nuclear magnetic resonance (^1^H-NMR, 400 MHz) and carbon-13 NMR spectra were measured with Varian Unity-400 spectrometers. Carbon multiplicities were obtained from DEPT experiments. Chemical shifts (δ) and coupling constants (Hz) were measured with respect to TMS or chloroform-*d*_1_. Mass and high-resolution mass spectra (HRMS) were taken on a Hewlett-Packard 5989A or JEOL JMS D-100 instrument, with a direct inlet system.

### 3.2. General Procedure

A mixture of 1,2,3,4-tetrahydrocarbazole (**1a**, 1 mmol), allyl acetate (**2a**, 2 mmol), Pt(acac)_2_ (9.7 mg, 0.025 mmol), and (4-ClC_6_H_4_)_3_P (36.5 mg, 0.1 mmol) in benzene (5 mL) was refluxed for 24 h. After cooling, the solvent was distilled under reduced pressure. Column chromatography (*n*-hexane/EtOAc = 4:1) of the residue afforded 146 mg (69%) of **3a** and 63 mg (30%) **4a**, respectively.

*N-Allyl-1,2,3,4-tetrahydrocarbazole* (**3a**) [62]: yellow oil. IR (KBr): *ν* 1644, 1613, 1464 cm^−1^. ^1^H-NMR (400 MHz, CDCl_3_): δ 7.47 (d, *J* = 7.6 Hz, 1H, ArH), 7.22 (d, *J* = 8.4 Hz, 1H, ArH), 7.12 (ddd, *J* = 8.4, 6.8, 0.8 Hz, 1H, ArH), 7.06 (ddd, *J* = 8.4, 6.8, 0.8 Hz, 1H, ArH), 5.90 (ddt, *J* = 17.2, 10.4, 4.8 Hz, 1H, vinyl H), 5.09 (ddt, *J* = 10.4, 1.6, 1.6 Hz, 1H, vinyl H), 4.88 (ddt, *J* = 17.2, 1.6, 1.6 Hz, 1H, vinyl H), 4.62 (dt, *J* = 4.8, 1.6 Hz, 2H, NCH_2_), 2.73 (tt, *J* = 6.0, 1.6 Hz, 2H, CH_2_), 2.67 (tt, *J* = 6.0, 1.6 Hz, 2H, CH_2_), 1.82–1.96 (m, 4H, CH_2_ × 2); ^13^C-NMR (100 MHz, CDCl_3_): δ 136.2 (C), 135.4 (C), 133.8 (CH), 127.4 (C), 120.5 (CH), 118.7 (CH), 117.7 (CH), 116.0 (CH_2_), 109.5 (C), 108.8 (CH), 45.0 (CH_2_), 23.2 (CH_2_), 23.2 (CH_2_), 22.0 (CH_2_), 21.1 (CH_2_). EI-MS: *m*/*z* 211 (M^+^), 196, 183, 168, 154, 142, 128, 115, 89, 77, 63, 51. EI-HRMS calcd. for C_15_H_17_N: 211.1361. Found: 211.1363.

*4a-Allyl-2,3,4,4a-tetrahydro-1H-carbazole* (**4a**) [62]: yellow oil. IR (KBr): *ν* 1711, 1641, 1617, 1451 cm^−1^. ^1^H-NMR (400 MHz, CDCl_3_): δ 7.58 (d, *J* = 7.6 Hz, 1H, ArH), 7.31 (ddd, *J* = 7.6, 7.2, 1.2 Hz, 1H, ArH), 7.30 (dd, *J* = 7.6, 1.2 Hz, 1H, ArH), 7.17 (ddd, *J* = 7.6, 7.2, 1.2 Hz, 1H, ArH), 5.17 (ddt, *J* = 17.2, 10.0, 6.8 Hz, 1H, vinyl H), 4.94 (ddt, *J* = 17.2, 2.0, 1.2 Hz, 1H, vinyl H), 4.86 (ddt, *J* = 10.0, 2.0, 1.2 Hz, 1H, vinyl H), 2.85–2.91 (m, 1H, CH), 2.62 (dt, *J* = 13.2, 6.4 Hz, 1H, CH), 2.54–2.59 (m, 1H, CH), 2.54 (dt, *J* = 13.2, 5.6 Hz, 1H, CH) 2.36 (ddt, *J* = 13.2, 3.2, 2.8 Hz, 1H, CH), 2.18–2.24 (m, 1H, CH), 1.83 (tq, *J* = 13.6, 4.0 Hz, 1H, CH), 1.66–1.72 (m, 1H, CH), 1.43 (tq, *J* = 13.6, 4.0 Hz, 1H, CH), 1.16 (dt, *J* = 13.6, 4.0 Hz, 1H, CH); ^13^C-NMR (100 MHz, CDCl_3_): δ 188.9 (C), 154.8 (C), 144.6 (C), 132.1 (CH), 127.6 (CH), 124.7 (CH), 121.9 (CH), 120.1 (CH), 118.0 (CH_2_), 57.6 (C), 37.6 (CH_2_), 37.0 (CH_2_), 30.1 (CH_2_), 28.8 (CH_2_), 21.1 (CH_2_). EI-MS: *m*/*z* 211 (M^+^), 196, 183, 170, 168, 154, 142, 128, 115, 89, 77, 63, 51. EI-HRMS calcd. for C_15_H_17_N: 211.1361. Found: 211.1358.

*N-(But-2-en-1-yl)-1,2,3,4-tetrahydrocarbazole* (**3b**): yellow oil. IR (KBr): *ν* 1653, 1611, 1462 cm^−1^. ^1^H-NMR (400 MHz, CDCl3): δ 7.46 (dd, *J* = 8.0, 1.2 Hz, 1H, ArH), 7.24 (dd, *J* = 8.0, 1.2 Hz, 1H, ArH), 7.12 (ddd, *J* = 8.0, 7.2, 1.2 Hz, 1H, ArH), 7.05 (ddd, *J* = 8.0, 7.2, 1.2 Hz, 1H, ArH), 5.40–5.61 (m, 2H, vinyl H), 4.53–4.55 (m, 2H, NCH_2_), 2.73 (tt, *J* = 6.0, 1.6 Hz, 2H, CH_2_), 2.68 (tt, *J* = 6.0, 1.6 Hz, 2H, CH_2_), 1.80–1.95 (m, 4H, CH_2_ × 2), 1.63 (dd, *J* = 6.4, 1.2 Hz, 3H, CH_3_); ^13^C-NMR (100 MHz, CDCl_3_): δ 136.0 (C), 135.3 (C), 127.3 (C), 126.7 (CH), 120.4 (CH), 118.6 (CH), 117.7 (CH), 109.3 (C), 108.9 (CH), 44.4 (CH_2_), 23.3 (CH_2_), 23.2 (CH_2_), 22.1 (CH_2_), 21.1 (CH_2_), 17.5 (CH_3_). EI-MS: *m*/*z* 225 (M^+^), 210, 197, 182, 168, 154, 143, 128, 115, 89, 77, 63, 51. EI-HRMS calcd. for C_16_H_19_N: 225.1517. Found: 225.1517.

*4a-(But-2-en-1-yl)-2,3,4,4a-tetrahydro-1H-carbazole* (**4b**) [16]: yellow oil. IR (KBr): *ν* 1711, 1616, 1584, 1450 cm^−1^. ^1^H-NMR (400 MHz, CDCl_3_): δ 7.57 (d, *J* = 7.6 Hz, 1H, ArH), 7.26–7.34 (m, 2H, ArH), 7.17 (t, *J* = 7.6 Hz, 1H, ArH), 5.37 (dq, *J* = 13.6, 6.4 Hz, 1H, vinyl H), 4.83 (tq, *J* = 13.6, 6.4 Hz, 1H, vinyl H), 2.84–2.89 (m, 1H, CH), 2.55–2.61 (m, 1H, CH) 2.54 (dt, *J* = 13.6, 5.6 Hz, 1H, CH) 2.44 (dd, *J* = 13.6, 7.6 Hz, 1H, CH) 2.34 (dq, *J* = 13.6, 2.8 Hz, 1H, CH), 2.15–2.24 (m, 1H, CH), 1.81 (tq, *J* = 13.6, 4.0 Hz, 1H, CH), 1.64–1.69 (m, 1H, CH), 1.48 (dd, *J* = 6.4, 0.8 Hz, 3H, CH_3_), 1.41 (tq, *J* = 13.6, 4.0 Hz, 1H, CH), 1.12 (dt, *J* = 13.6, 4.0 Hz, 1H, CH); ^13^C-NMR (100 MHz, CDCl_3_): δ 189.2 (C), 154.8 (C), 144.9 (C), 128.6 (CH), 127.4 (CH), 124.5 (CH), 124.4 (CH), 121.9 (CH), 120.0 (CH), 57.8 (C), 36.7 (CH_2_), 36.4 (CH_2_), 30.1 (CH_2_), 28.8 (CH_2_), 21.0 (CH_2_), 17.7 (CH_3_). EI-MS: *m*/*z* 225 (M^+^), 210, 196, 182, 168, 154, 143, 128, 115, 89, 77, 63. EI-HRMS calcd. for C_16_H_19_N: 225.1517. Found: 225.1518.

*N-(Hex-2-en-1-yl)-1,2,3,4-tetrahydrocarbazole* (**3c**): yellow oil. IR (KBr): *ν* 1658, 1613, 1464 cm^−1^. ^1^H-NMR (400 MHz, CDCl_3_): δ 7.54 (d, *J* = 7.6 Hz, 1H, ArH), 7.32 (d, *J* = 8.0 Hz, 1H, ArH), 7.20 (dt, *J* = 8.0, 1.2 Hz, 1H, ArH), 7.13 (dt, *J* = 7.6, 1.2 Hz, 1H, ArH), 5.47–5.61 (m, 2H, vinyl H), 4.63 (dd, *J* = 4.8, 1.2 Hz, 2H, NCH_2_), 2.81 (t, *J* = 6.0 Hz, 2H, CH_2_), 2.75 (t, *J* = 6.0 Hz, 2H, CH_2_), 1.90–2.05 (m, 6H, CH_2_ × 3), 1.41 (hext, *J* = 7.6 Hz, 2H, CH_2_), 0.92 (t, *J* = 7.6 Hz, 3H, CH_3_); ^13^C-NMR (100 MHz, CDCl_3_): δ 136.1 (C), 135.3 (C), 132.6 (CH), 127.3 (C), 125.6 (CH), 120.4 (CH), 118.5 (CH), 117.6 (CH), 109.3 (C), 108.9 (CH), 44.6 (CH_2_), 36.5 (CH_2_), 23.3 (CH_2_), 23.2 (CH_2_), 22.2 (CH_2_), 22.1 (CH_2_), 21.1 (CH_2_), 13.6 (CH_3_). EI-MS: *m*/*z* 253 (M^+^), 225, 210, 196, 182, 168, 154, 143, 128, 115, 89, 77, 63, 55. EI-HRMS calcd. for C_18_H_23_N: 253.1830. Found: 253.1830.

*4a-(Hex-2-en-1-yl)-2,3,4,4a-tetrahydro-1H-carbazole* (**4c**): yellow oil. IR (KBr): *ν* 1711, 1616, 1586, 1463 cm^−^^1^. ^1^H-NMR (400 MHz, CDCl_3_): δ 7.57 (d, *J* = 7.6 Hz, 1H, ArH), 7.31 (dt, *J* = 7.6, 1.2 Hz, 1H, ArH), 7.28 (dd, *J* = 7.6, 1.2 Hz, 1H, ArH), 7.17 (dt, *J* = 7.6, 1.2 Hz, 1H, ArH), 5.33 (dt, *J* = 15.2, 6.8 Hz, 1H, vinyl H), 4.81 (dt, *J* = 15.2, 6.8 Hz, 1H, vinyl H), 2.85–2.90 (m, 1H, CH), 2.53–2.59 (m, 2H, CH_2_), 2.49 (dt, *J* = 13.6, 7.6 Hz, 1H, CH), 2.35 (dq, *J* = 13.6, 2.8 Hz, 1H, CH), 2.16–2.23 (m, 1H, CH), 1.65–1.88 (m, 4H, CH × 4), 1.43 (tq, *J* = 13.6, 4.4 Hz, 1H, CH), 1.15–1.23 (m, 2H, CH_2_), 1.14 (dt, *J* = 13.6, 4.4 Hz, 1H, CH), 0.73 (t, *J* = 7.2, Hz, 3H, CH_3_); ^13^C-NMR (100 MHz, CDCl_3_): δ 189.2 (C), 154.8 (C), 144.9 (C), 134.2 (CH), 127.4 (CH), 124.5 (CH), 123.3 (CH), 121.9 (CH), 120.0 (CH), 57.9 (C), 36.7 (CH_2_), 36.4 (CH_2_), 34.3 (CH_2_), 30.1 (CH_2_), 28.8 (CH_2_), 22.4 (CH_2_) 21.1 (CH_2_), 13.4 (CH_3_). EI-MS: *m*/*z* 253 (M^+^), 225, 210, 196, 182, 168, 154, 143, 128, 115, 89, 77, 63, 55. EI-HRMS calcd. for C_18_H_23_N: 253.1830. Found: 253.1832.

*N-Allyl-6,7,8,9,10,10a-hexahydro-cyclohepta[b]indole* (**3d**): yellow oil. IR (KBr): *ν* 1643, 1611, 1465 cm^−1^. ^1^H-NMR (400 MHz, CDCl_3_): δ 7.56 (dd, *J* = 7.6 Hz, 1.2, 1H, ArH), 7.25 (dd, *J* = 7.2, 1.2 Hz, 1H, ArH), 7.17 (dt, *J* = 7.2, 1.2 Hz, 1H, ArH), 7.13 (dt, *J* = 7.2, 1.2 Hz, 1H, ArH), 5.97 (ddt, *J* = 17.2, 10.4, 4.4 Hz, 1H, vinyl H), 5.14 (ddt, *J* = 10.4, 2.0, 1.2 Hz, 1H, vinyl H), 4.88 (ddt, *J* = 17.2, 2.0, 1.2 Hz, 1H, vinyl H), 4.73 (dt, *J* = 4.4, 2.0 Hz, 2H, NCH_2_), 2.84–2.93 (m, 4H, CH_2_ × 2), 1.80–1.98 (m, 6H, CH_2_ × 3); ^13^C-NMR (100 MHz, CDCl_3_): δ 138.8 (C), 135.3 (C), 134.0 (CH), 127.9 (C), 120.3 (CH), 118.7 (CH), 117.6 (CH), 115.8 (CH_2_), 113.9 (C), 108.8 (CH), 45.0 (CH_2_), 31.7 (CH_2_), 28.4 (CH_2_), 27.2 (CH_2_), 26.3 (CH_2_) 24.4 (CH_2_). EI-MS: *m*/*z* 225 (M^+^), 209, 196, 182, 168, 156, 142, 128, 115, 89, 77, 63, 51. EI-HRMS calcd. for C_16_H_19_N: 225.1517. Found: 225.1517.

*10a-Allyl-6,7,8,9,10,10a-hexahydrocyclohepta[b]indole* (**4d**) [16]: yellow oil. IR (KBr): *ν* 1710, 1622, 1469 cm^−1^. ^1^H-NMR (400 MHz, CDCl_3_): δ 7.50 (d, *J* = 8.0 Hz, 1H, ArH), 7.29 (t, *J* = 8.0 Hz, 1H, ArH), 7.22 (d, *J* = 8.0 Hz, 1H, ArH), 7.18 (t, *J* = 8.0 Hz, 1H, ArH), 5.21 (ddt, *J* = 16.8, 10.0, 6.8 Hz, 1H, vinyl H), 4.91 (d, *J* = 16.8 Hz, 1H, vinyl H), 4.85 (d, *J* = 10.0 Hz, 1H, vinyl H), 2.92 (ddd, *J* = 13.6, 6.0, 4.0 Hz, 1H, CH), 2.56–2.64 (m, 2H), 2.45 (dd, *J* = 13.6, 7.6, 1H, CH), 1.97–2.09 (m, 2H, CH_2_), 1.70–1.82 (m, 2H, CH_2_), 1.54–1.65 (m, 2H, CH_2_), 1.41–1.51 (m, 1H, CH), 0.67–0.76 (m, 1H, CH); ^13^C-NMR (100 MHz, CDCl_3_): δ 190.7 (C), 154.6 (C), 143.5 (C), 132.2 (CH), 127.6 (CH), 124.9 (CH), 121.8 (CH) 119.6 (CH), 117.9 (CH_2_), 62.1 (C), 41.5 (CH), 34.9 (CH_2_), 31.3 (CH_2_), 30.4 (CH_2_), 28.5 (CH_2_), 24.5 (CH_2_). EI-MS: *m*/*z* 225 (M^+^), 210, 196, 184, 168, 156, 143,128, 115, 89, 77, 63, 51. EI-HRMS calcd. for C_16_H_19_N: 225.1517. Found: 225.1516.

*N-Allyl-2,3-dimethylindole* (**3e**) [63]: yellow oil. IR (KBr): *ν* 1696, 1614, 1452 cm^−1^. ^1^H-NMR (400 MHz, CDCl_3_): δ 7.49 (dd, *J* = 7.6, 1.2 Hz, 1H, ArH), 7.20 (dd, *J* = 7.6, 1.2 Hz, 1H, ArH), 7.12 (ddd, *J* = 7.6, 7.2, 1.2 Hz, 1H, ArH), 7.07 (ddd, *J* = 7.6, 7.2, 1.2 Hz, 1H, ArH), 5.92 (ddt, *J* = 17.2, 10.4, 4.4 Hz, 1H, vinyl H), 5.09 (ddt, *J* = 10.4, 2.0, 1.2 Hz, 1H, vinyl H), 4.82 (ddt, *J* = 17.2, 2.0, 1.2 Hz, 1H, vinyl H), 4.66 (dt, *J* = 4.4, 2.0 Hz, 2H, CH_2_), 2.31 (s, 3H, CH_3_), 2.26 (s, 3H, CH_3_); ^13^C-NMR (100 MHz, CDCl_3_): δ 136.0 (C), 133.7 (CH), 132.2 (C), 128.5 (C), 120.5 (CH), 118.7 (CH), 117.9 (CH), 115.9 (CH_2_), 108.6 (CH), 106.7 (C), 45.3 (CH_2_), 9.9 (CH_3_), 8.8 (CH_3_). EI-MS: *m*/*z* 185 (M^+^), 170, 158, 144, 128, 115, 102, 88, 77, 51. EI-HRMS calcd. for C_13_H_15_N: 185.1204. Found: 185.1202.

*3-Allyl-2,3-dimethyl-3H-indole* (**4e**) [16]: yellow oil. IR (KBr): *ν* 1712, 1638, 1604, 1453 cm^−1^. ^1^H-NMR (400 MHz, CDCl_3_): δ 7.50 (dd, *J* = 7.6, 1.2 Hz, 1H, ArH), 7.28 (dd, *J* = 7.6, 1.2 Hz, 1H, ArH), 7.23–7.26 (m, 1H, ArH), 7.16 (ddd, *J* = 7.6, 7.2, 1.2 Hz, 1H, ArH), 5.13 (ddt, *J* = 17.2, 10.4, 6.4 Hz, 1H, vinyl H), 4.92 (ddt, *J* = 17.2, 2.0, 1.2 Hz, 1H, vinyl H), 4.83 (ddt, *J* = 10.0, 2.0, 1.2 Hz, 1H, vinyl H), 2.60 (ddt, *J* = 13.6, 6.4, 1.2 Hz, 1H, CH), 2.38 (ddt, *J* = 13.6, 8.0, 1.2 Hz, 1H, CH), 2.33 (s, 3H, CH_3_), 1.28 (s, 3H, CH_3_); ^13^C-NMR (100 MHz, CDCl_3_): δ 186.4 (C), 154.1 (C), 143.3 (C), 132.4 (CH), 127.6 (CH), 124.9 (CH), 121.7 (CH), 119.7 (CH), 117.9 (CH_2_), 57.4 (CH_2_), 41.1 (CH_2_), 21.7 (CH_3_), 15.8 (CH_3_). EI-MS: *m*/*z* 185 (M^+^), 170, 158, 144, 128, 115, 102, 88, 77, 51.EI-HRMS calcd. for C_13_H_15_N: 185.1204. Found: 185.1202.

*N-Allyl-3-methylindole* (**3f**) [64]: yellow oil. IR (KBr): *ν* 1614, 1459 cm^−1^. ^1^H-NMR (400 MHz, CDCl_3_): δ 7.57 (ddd, *J* = 8.4, 1.2, 0.8 Hz, 1H, ArH), 7.27 (ddd, *J* = 8.0, 1.2, 0.8 Hz, 1H, ArH), 7.18 (ddd, *J* = 8.4, 6.8, 1.2 Hz, 1H, ArH), 7.10 (ddd, *J* = 8.0, 6.8, 0.8 Hz, 1H, ArH), 6.86 (s, *J* = 1 Hz, ArH), 5.96 (ddt, *J* = 17.2, 10.0, 5.2 Hz, 1H, vinyl H), 5.16 (ddt, *J* = 10.0, 1.6, 1.2 Hz, 1H, vinyl H), 5.07 (ddt, *J* = 17.2, 1.6, 1.2 Hz, 1H, vinyl H), 4.65 (dt, *J* = 5.2, 1.6 Hz, 2H, NCH_2_), 2.32 (s, 3H, CH_3_); ^13^C-NMR (100 MHz, CDCl_3_): δ 136.4 (C), 133.8 (CH), 128.9 (C), 125.4 (CH), 121.4 (CH), 119.0 (CH), 118.6 (CH), 117.0 (CH_2_), 110.5 (C), 109.3 (CH), 48.5 (CH_2_), 9.6 (CH_3_). EI-MS: *m*/*z* 171 (M^+^), 156, 144, 130, 129, 103, 89, 77, 51. EI-HRMS calcd. for C_12_H_13_N: 171.1048. Found: 171.1051.

*3-Allyl-3-methyl-3H-indole* (**4f**) [16]: yellow oil. IR (KBr): *ν* 1711, 1638, 1603, 1479 cm^−1^. ^1^H-NMR (400 MHz, CDCl_3_): δ 8.03 (s, 1H, CH=N), 7.63 (dd, *J* = 7.6, 1.2 Hz, 1H, ArH), 7.34 (dt, *J* = 7.6, 1.2 Hz, 1H, ArH), 7.31 (dd, *J* = 7.2, 1.2 Hz, 1H, ArH), 7.26 (ddd, *J* = 7.6, 7.2, 1.2 Hz, 1H, ArH), 5.51 (ddt, *J* = 17.2, 10.0, 7.2 Hz, 1H, vinyl H), 5.02 (ddt, *J* = 17.2, 2.0, 1.2 Hz, 1H, vinyl H), 4.98 (ddt, *J* = 10.0, 2.0, 1.2 Hz, 1H, vinyl H), 2.49 (dt, *J* = 7.2, 1.2 Hz, 1H, CH), 2.48 (dt, *J* = 7.2, 1.2 Hz, 1H, CH), 1.35 (s, 3H, CH_3_); ^13^C-NMR (100 MHz, CDCl_3_): δ 178.9 (CH), 154.8 (C), 143.2 (C), 132.8 (CH), 127.8 (CH), 126.1 (CH), 121.7 (CH), 121.2 (CH), 118.6 (CH_2_), 57.0 (C), 40.2 (CH_2_), 19.6 (CH_3_). EI-MS: *m*/*z* 170 (M^+^), 156, 144, 128, 115, 103, 77, 51. EI-HRMS calcd. for C_12_H_13_N: 171.1048. Found: 171.1047.

## 4. Conclusions

In conclusion, we developed a catalytic system that used platinum-catalyzed allylation of heterocycle fused indoles with allylic acetates for a convenient and simple method to form C–N or C–C bonds. The reaction condition did not occur without any platinum catalyst and phosphine ligand. The allylation of allylic acetates worked well with carbazoles, giving generally good yields of corresponding allylic carbazoles.

## Figures and Tables

**Table 1:** Reaction of 1,2,3,4-tetrahydrocarbazole (**1a**) with allyl acetate (**2a**). ^a^

Entry	Ligand	Platinum Catalyst	Solvent	Yield (%) (3a:4a) ^b^
1	PPh_3_	Pt(acac)_2_	Benzene	7 (7:0)
2	(2-CH_3_C_6_H_4_)_3_P	Pt(acac)_2_	Benzene	7 (0:7)
3	(3-CH_3_C_6_H_4_)_3_P	Pt(acac)_2_	Benzene	3 (0:3)
4	(4-CH_3_C_6_H_4_)_3_P	Pt(acac)_2_	Benzene	14 (8:6)
5	(4-FC_6_H_4_)_3_P	Pt(acac)_2_	Benzene	11 (0:11)
6	(4-ClC_6_H_4_)_3_P	Pt(acac)_2_	Benzene	99 (69:30)
7	(*n*-butyl)_3_P	Pt(acac)_2_	Benzene	30 (0:30)
8	(3-CH_3_OC_6_H_4_)_3_P	Pt(acac)_2_	Benzene	11 (3:8)
9	(4-CH_3_OC_6_H_4_)_3_P	Pt(acac)_2_	Benzene	18 (4:14)
10	(2-furyl)_3_P	Pt(acac)_2_	Benzene	8 (0:8)
11	(2,6-diCH_3_OC_6_H_3_)_3_P	Pt(acac)_2_	Benzene	19 (9:10)
12	(2,4,6-triCH_3_OC_6_H_2_)_3_P	Pt(acac)_2_	Benzene	6 (0:6)
13	Dppm ^c^	Pt(acac)_2_	Benzene	9 (5:4)
14	Dppf ^d^	Pt(acac)_2_	Benzene	13 (2:11)
15	Dppe ^e^	Pt(acac)_2_	Benzene	14 (9:5)
16	Dppb ^f^	Pt(acac)_2_	Benzene	12 (5:7)
17	-	Pt(acac)_2_	Benzene	0 (0:0)
18	(4-ClC_6_H_4_)_3_P	-	Benzene	0 (0:0)
19 ^g^	(4-ClC_6_H_4_)_3_P	Pt(acac)_2_	Benzene	11 (5:6)
20 ^h^	(4-ClC_6_H_4_)_3_P	Pt(acac)_2_	Benzene	45 (32:13)
21	(4-ClC_6_H_4_)_3_P	*cis*-PtCl_2_(PhCN)_2_	Benzene	16 (6:10)
22	(4-ClC_6_H_4_)_3_P	*cis*-PtCl_2_(PPh_3_)_2_	Benzene	18 (5:13)
23	(4-ClC_6_H_4_)_3_P	Di(1,5-cyclooctadiene)Pt	Benzene	22 (7:15)
24	(4-ClC_6_H_4_)_3_P	O[Si(CH_3_)_2_C=CH_2_]_2_Pt	Benzene	12 (6:6)
25	(4-ClC_6_H_4_)_3_P	PtCl_2_	Benzene	36 (11:25)
26	(4-ClC_6_H_4_)_3_P	PtI_2_	Benzene	11 (0:11)
27	(4-ClC_6_H_4_)_3_P	Pt(CN)_2_	Benzene	20 (17:3)
28	-	Pt(CH_2_=CH_2_)(PPh_3_)_2_	Benzene	17 (2:15)
29	(4-ClC_6_H_4_)_3_P	Pt(CH_2_=CH_2_)(PPh_3_)_2_	Benzene	35 (5:30)
30	-	Pt(PPh_3_)_4_	Benzene	22 (2:20)
31	(4-ClC_6_H_4_)_3_P	Pt(PPh_3_)_4_	Benzene	64 (4:60)
32 ^i^	(4-ClC_6_H_4_)_3_P	Pt(acac)_2_	Benzene	0 (0:0)
33 ^j^	(4-ClC_6_H_4_)_3_P	Pt(acac)_2_	Benzene	11 (5:6)
34 ^k^	(4-ClC_6_H_4_)_3_P	Pt(acac)_2_	Benzene	75 (38:37)
35	(4-ClC_6_H_4_)_3_P	Pt(acac)_2_	Toluene	82 (62:20)
36	(4-ClC_6_H_4_)_3_P	Pt(acac)_2_	CH_2_Cl_2_	43 (40:3)
37	(4-ClC_6_H_4_)_3_P	Pt(acac)_2_	THF	52 (34:18)
38	(4-ClC_6_H_4_)_3_P	Pt(acac)_2_	Dioxane	78 (63:15)
39	(4-ClC_6_H_4_)_3_P	Pt(acac)_2_	DMF	67 (53:14)

^a^ Reaction conditions: **1a** (1 mmol), **2a** (2 mmol), Pt catalyst (0.025 mmol), and ligand (0.1 mmol) in solvent (5 mL) were refluxed for 24 h. ^b^ Isolated yield. ^c^ 1,1-Bis(diphenylphosphino)methane. ^d^ 1,1-Bis(diphenylphosphino)ferrocene. ^e^ 1,2-Bis(diphenylphosphino)ethane. ^f^ 1,4-Bis(diphenylphosphino)butane. ^g^ Stirred at 50 °C. ^h^ Refluxed for 12 h. ^i^ (4-ClC_6_H_4_)_3_P (0.025 mmol). ^j^ (4-ClC_6_H_4_)_3_P (0.05 mmol). ^k^ (4-ClC_6_H_4_)_3_P (0.075 mmol).

**Table 2:** Reaction of 1,2,3,4-tetrahydrocarbazole (**1a**) with allylic compounds (**2b**–**f**). ^a^

Entry	2	Yield ^b^ (%)	
1	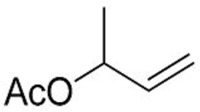 **2b**	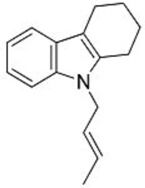 **3b** 54 (*E*/*Z* = 90/10) ^d^	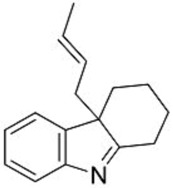 **4b** 23 (*E/Z* = 83/17) ^d^
2	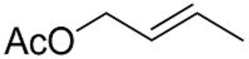 **2c**	**3b** 62 (*E/Z* = 89/11) ^d^	**4b** 25 (*E/Z* = 95/5) ^d^
3	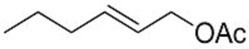 **2d**	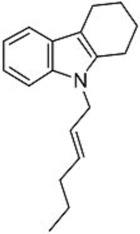 **3c** 45 (*E/Z* = 85/15) ^d^	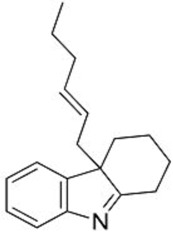 **4c** 27 (*E/Z* = 90/10) ^d^
4	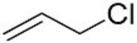 **2e**	**3a** 34	**4a** 12
5 ^c^	**2e**	**3a** 55	**4a** 20
6	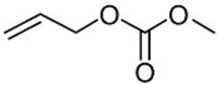 **2f**	**3a** 87	**4a** 5

^a^ Reaction conditions: **1a** (1 mmol), **2** (2 mmol), Pt(acac)_2_ (0.025 mmol), and (4-ClC_6_H_4_)_3_P (0.1 mmol) in benzene (5 mL) were refluxed for 24 h. ^b^ Isolated yield. ^c^ Pt(acac)_2_ (0.05 mmol) and (4-ClC_6_H_4_)_3_P (0.2 mmol). ^d^ Determined by GC.

**Table 3:** Allylation of indoles (**1**) with allyl acetate (**2a**). ^a^

Entry	1	Yield ^b^ (%)	
1	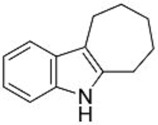 **1b**	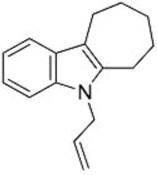 **3d** 67	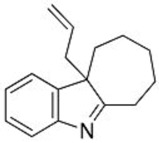 **4d** 30
2	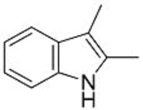 **1c**	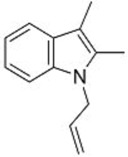 **3e** 68	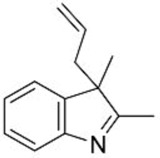 **4e** 30
3	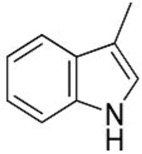 **1d**	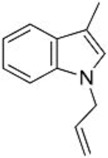 **3f** 47	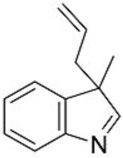 **4f** 24

^a^ Reaction conditions: **1** (1 mmol), **2a** (2 mmol), Pt(acac)_2_ (0.025 mmol), and (4-ClC_6_H_4_)_3_P (0.1 mmol) in benzene (5 mL) were refluxed for 24 h. ^b^ Isolated yield.
